# Supplementary material for: Dynamic co-catalysis of Au single atoms and nanoporous Au for methane pyrolysis
Source: Nat Commun. 2020 Apr 21;11:1919. doi: 10.1038/s41467-020-15806-8 (PMC7174348; doi:10.1038/s41467-020-15806-8)
Supplement: Supplementary file 1 — Supplementary Information [file 41467_2020_15806_MOESM1_ESM.pdf]

Supplementary Information

Dynamic co-catalysis of Au single atoms and nanoporous Au for methane pyrolysis

By Xi et al.

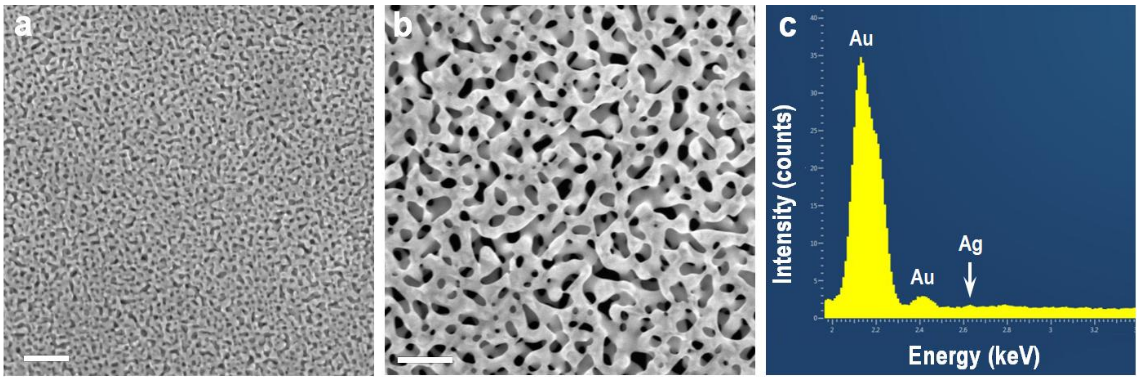

Supplementary Figure 1 SEM images (**a**,**b**) and EDS result (**c**) of as-prepared NPG. From 8 EDS measurements, the average Ag content is found to be 1.37 at%, and the corresponding standard deviation is 0.38 at%. Scale bar: **a**, 500 nm; **b**, 200 nm. The source data of 1.37 ± 0.38 at% are provided as a Source Data file.

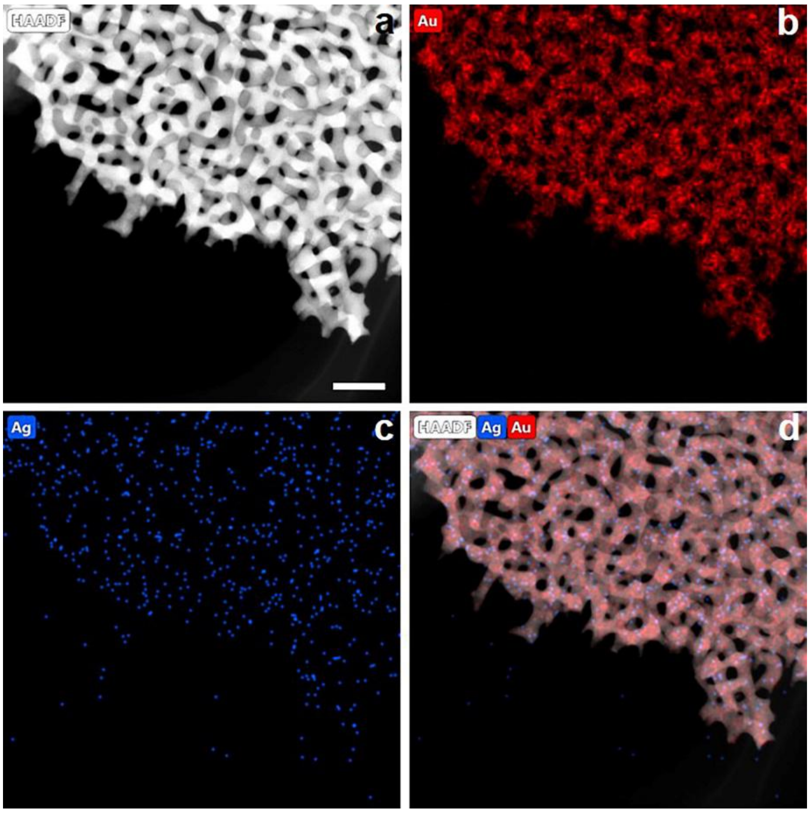

Supplementary Figure 2 EDS mapping of as-prepared NPG. **a** HAADF image. **b**,**c** Maps of Au and Ag. **d** Overlay of **a**–**c**. The images in **a**–**d** have the same scale bar of 200 nm.

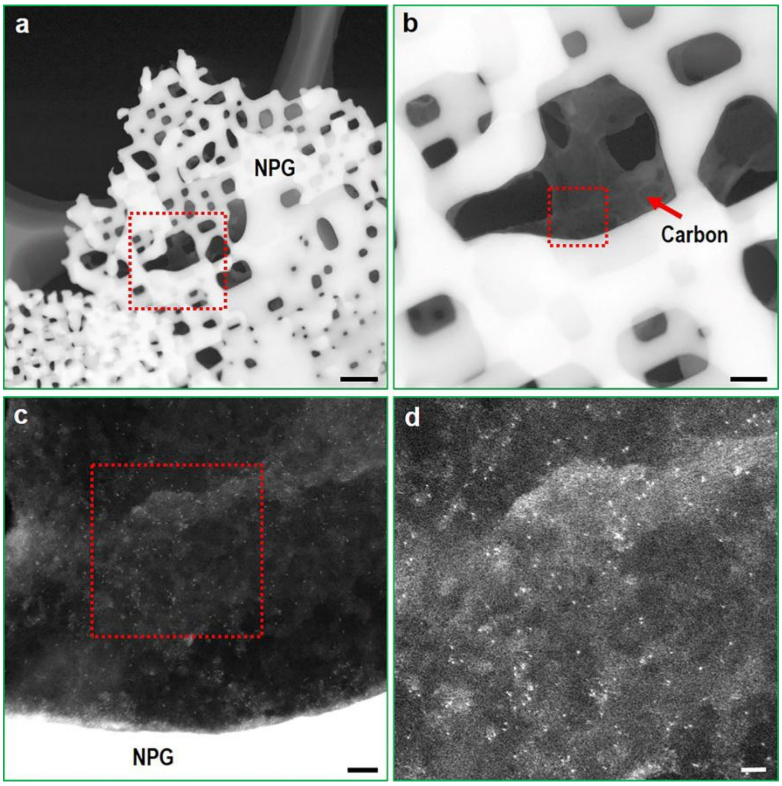

Supplementary Figure 3 HAADF images of an area with the boundary of NPG and carbon structure. **a**–**c** Low-, medium- and high-magnification HAADF images of the area. **d** Atomic-resolution HAADF image showing the Au single atoms in the carbon part. Panels **b**–**d** are taken from the boxed regions in **a**–**c**, respectively. Scale bars: **a**, 200 nm; **b**, 50 nm; **c**, 5 nm; **d**, 2 nm.

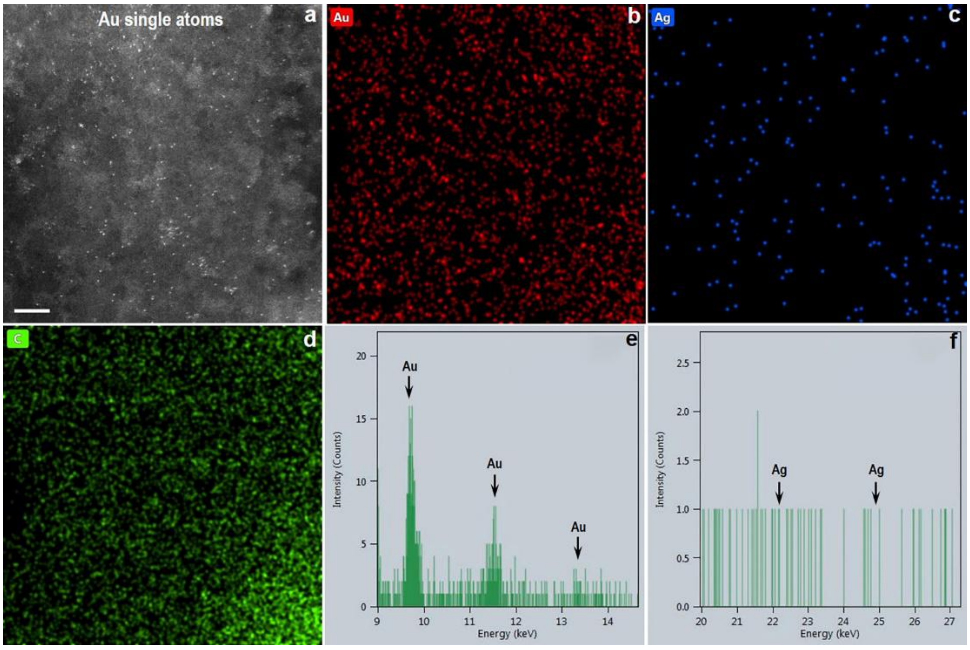

Supplementary Figure 4 EDS mapping of the Au single atoms in the carbon structure. **a** HAADF image of the Au single atoms. **b**–**d** EDS mapping images using the Au, Ag and C signals from the region in **a**. **e**,**f** EDS spectra from the region in **a**. The images in **a**–**d** have the same scale bar of 5 nm. The mapping results indicate that the distribution of the Au single atoms in the carbon structure is homogeneous. The Ag signals in **f** are below the detecting limit, and only noise exists in the Ag map in **c**, reasonably suggesting that there is no Ag in the carbon layer.

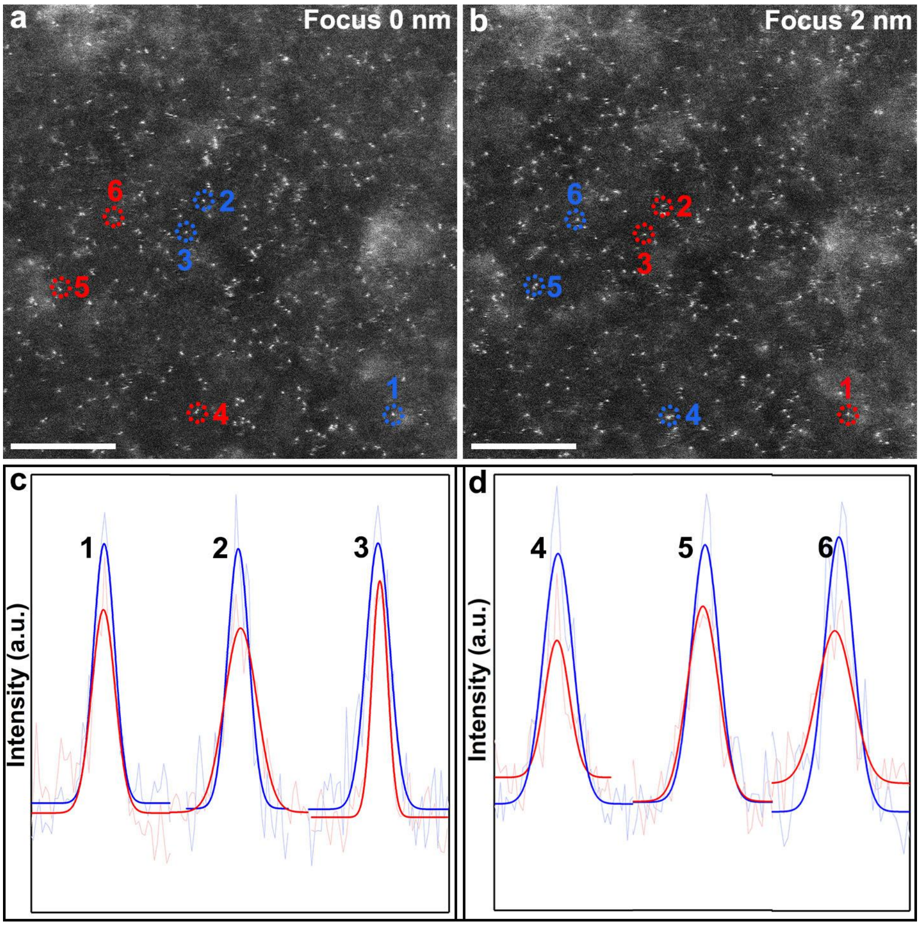

Supplementary Figure 5 Contrast intensity analysis of single atoms with different focus in amorphous carbon generated through methane pyrolysis. **a,b** HAADF images of the Au single atoms in amorphous carbon with focus on 0 nm and 2 nm (relative height), respectively. **c,d** The comparison of the same single atoms contrast profile in **a** and **b**. The contrast intensity of single atoms are sharper when they are near the focal plane. Whereas, their contrast intensities become lower under defocus conditions. The blue circles indicate the brighter single atoms, and the red circles indicates the lower bright single atoms in **a** and **b**, respectively. Scale bars: **a**, 5 nm; **b**, 5 nm.

Supplementary Note 1 In order to investigate the depth distribution of the single atoms, we changed the focal plane and took HAADF images at different depth, which are shown in

Supplementary Fig. 5. Indeed, the contrast intensity analyses of the single atoms with different focus conditions show that their intensities are highest when they are near the focal plane; whereas, they become comparably lower under defocus conditions. Comparing with Supplementary Fig. 5**a** and **b**, some single atoms are bright at focus 0 nm and less bright at focus 2 nm (as indicated by circles 1–3 and Supplementary Fig. 5**c**). And another single atoms are inverse (as indicated by circles 4–6 and Supplementary Fig. 5**d**). Therefore, it can be speculated that single atoms (Au or possible Ag) are distributed in the whole amorphous carbon generated from the pyrolysis of methane.

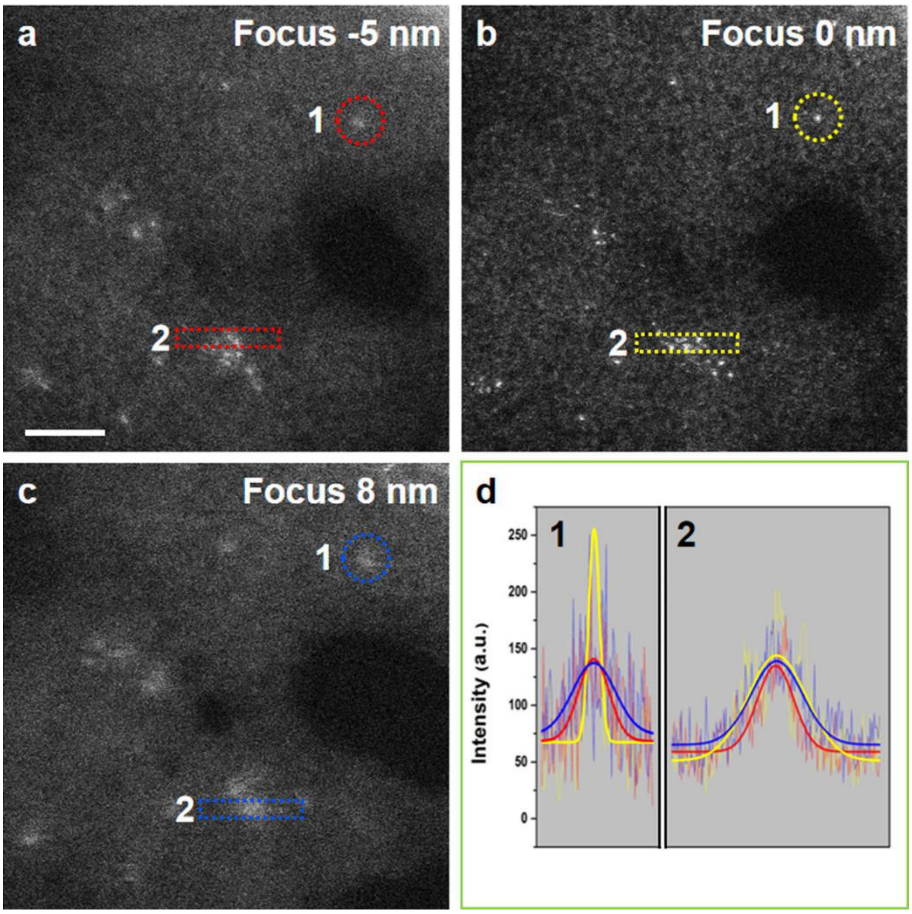

Supplementary Figure 6 Contrast intensity analysis of stable single atoms and white patchy areas under different focus conditions. **a**–**c** HAADF images of the same Au single atoms and white patchy areas in amorphous carbon with focus on -5 nm, 0 nm and 8 nm (relative height), respectively. **d** The compared contrast profile of the same single atoms and white patchy areas in **a, b** and **c**, as indicated by circles 1 and boxes 2, respectively. Scale bars: **a**, 2 nm; **b**, 2 nm; **c**, 2 nm.

Supplementary Note 2 It should be pointed out that during the process of taking HAADF images, the position of some single atoms may change (Supplementary Fig. 5**a**, **b** and Supplementary Note 1). The sample area shown in Supplementary Fig. 6 represents a relatively stable region, which provides us more accurate contrast intensity information for individual atoms. Again, single atoms and white patchy areas are sharper when they are in focus plane, while their intensities become weaker upon focus change.

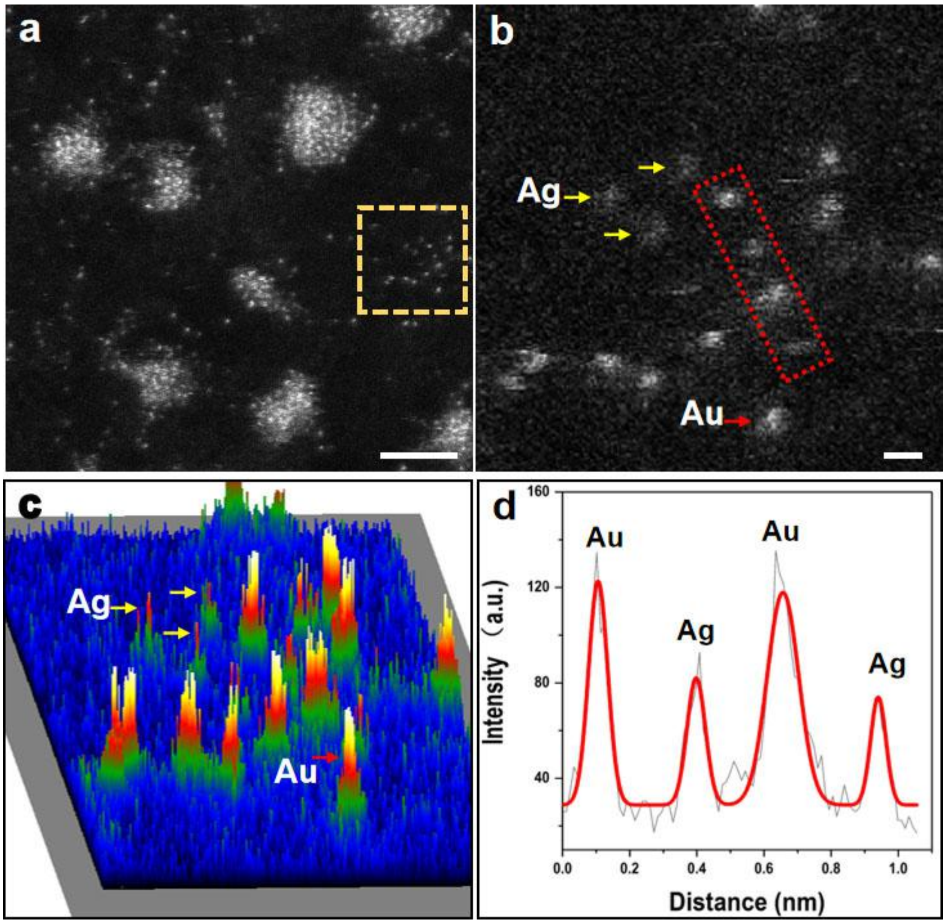

Supplementary Figure 7 Contrast intensity comparison of Au and Ag single atoms co-existing on an ultra-thin carbon film. **a** HAADF image showing Au and Ag single atoms and clusters. They were produced using the magnetron sputtering technique. Because the single atoms are all located on the surface of the ultra-thin carbon film, we can consider that in a small localized area, the single atoms are approximate all at a same plane. **b** Enlarged image of the single atoms in the box in **a**. It can be clearly seen that the contrasts of Au and Ag single atoms are different. **c** 3D contrast intensity analysis of **b**. **d** Contrast intensity profile in the dashed box in **b**, where the contrast of Au single atoms is obvious higher than that of Ag. Scale bars: **a**, 2 nm; **b**, 0.2 nm.

Supplementary Note 3 The contrast intensity of a single atom is related to its distance from the focal plane (Supplementary Figs. 5, 6 and Supplementary Notes 1, 2). We cannot simply assume that

certain bright spots with lower intensities in Fig. 1**f** are Ag single atoms. We supplemented some experiments to analyze the contrast difference between Au and Ag single atoms in a same focal plane. Firstly, we used the magnetron sputtering technique to prepare a new sample with Au and Ag single atoms co-existing on the surface of an ultra-thin carbon film on a Cu grid (Beijing XXBR Technology Co., Ltd), and imaged them with HAADF (Supplementary Fig. 7**a**, **b**). From the contrast intensity analysis results shown in Supplementary Figs. 7c, d, it can be seen that the Au single atoms have higher contrast intensities than the Ag, and their contrast intensities are both obviously higher than that of carbon.

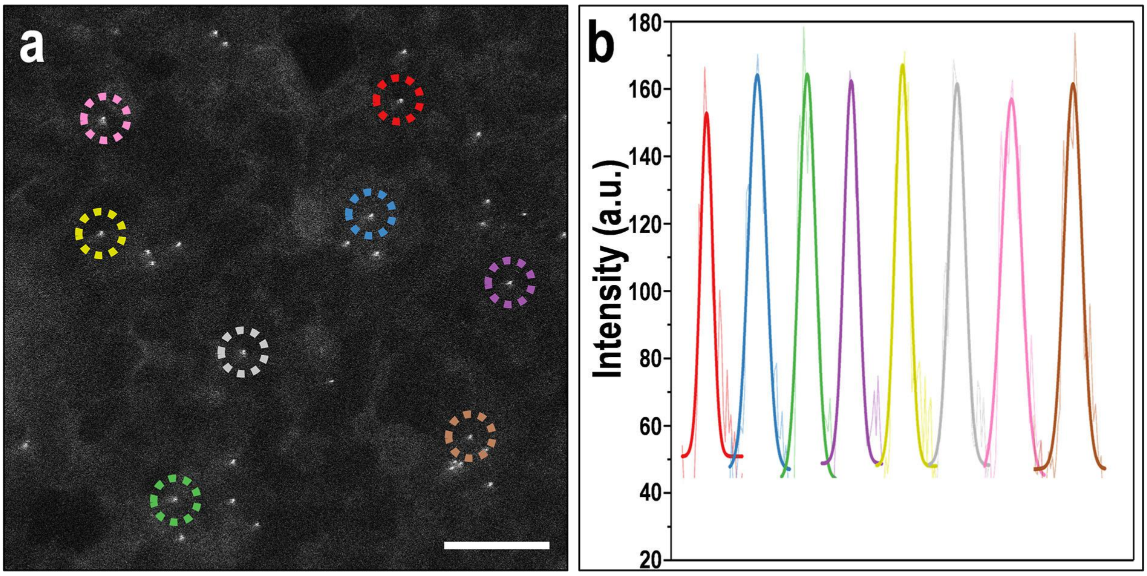

Supplementary Figure 8 Contrast intensity analysis of single atoms in thin amorphous carbon generated through methane pyrolysis. **a** HAADF image of Au single atoms in ultrathin amorphous carbon. **b** The comparison chart of the single atoms contrast intensity randomly selected in **a**. It can be seen from **b** that there is no significant difference in the contrast of those single atoms. Thus, it can be speculated that widely distributed and uniform Au single atoms have formed during the methane pyrolysis. Scale bars: **a**, 5 nm.

Supplementary Note 4 Based on this contrast intensity analyses results of Au and Ag single atoms in Supplementary Fig. 7 and Supplementary Note 3, we analyzed the contrast information of single atoms in thin amorphous carbon generated during the methane pyrolysis process, to further distinguish single atoms species. The results show that the contrast intensity of single atoms in amorphous carbon is relative uniform, and there is almost no difference in their contrast profile (Supplementary Fig. 8**b**). And no Ag signals were detected in the amorphous carbon according to EDS results (Supplementary Figs. 4 and 11). Therefore, it can be determined that widely distributed and uniform Au single atoms have formed during methane pyrolysis.

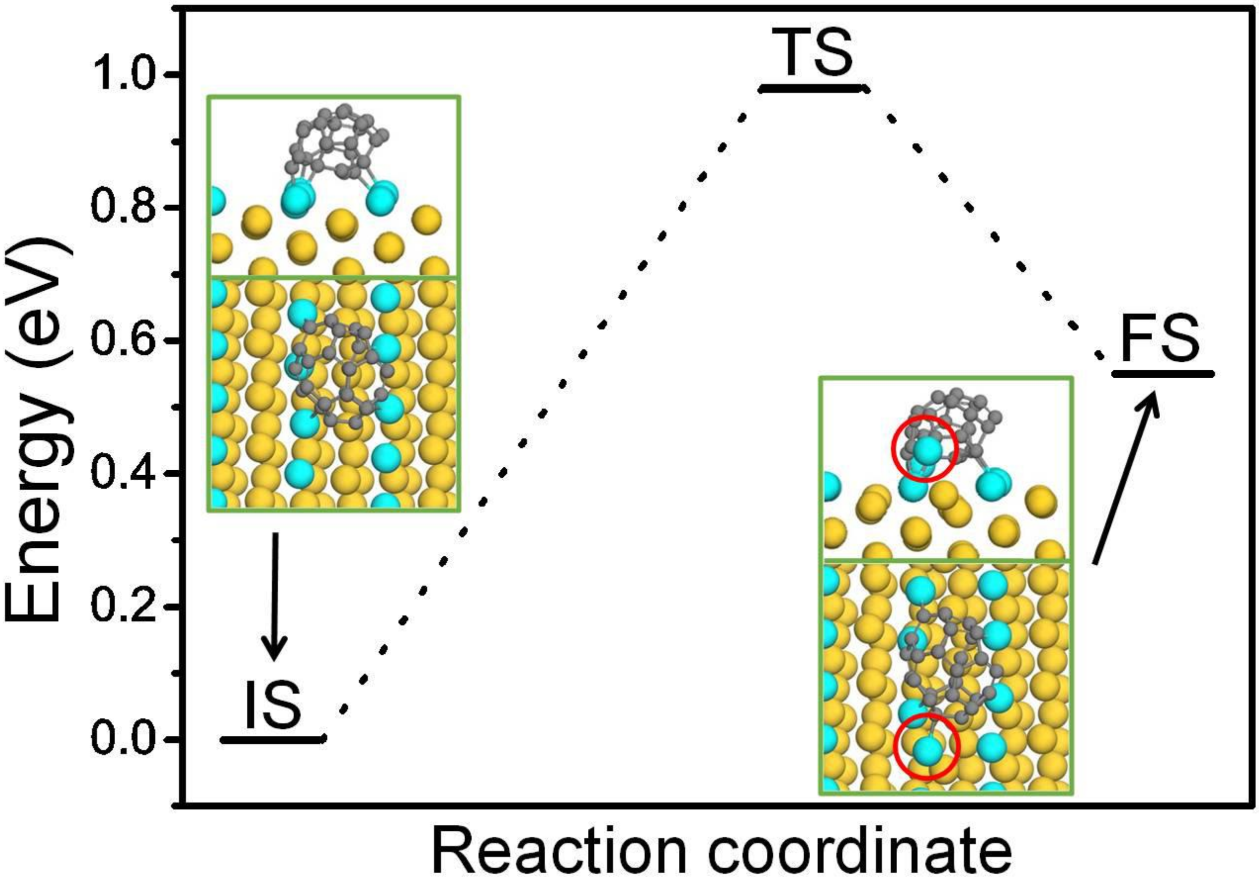

Supplementary Figure 9 DFT result of the configuration evolution of an amorphous carbon cluster adsorbed on the surface of an Au crystal. The initial state (IS) of this configuration can reach a final state (FS) through a transition state (TS). The left and the right insets are the DFT-relaxed atomic configurations of the IS and the FS, respectively. Each of the insets contains two panels, of which the top and the bottom panels are the side and the top views of the corresponding configuration, respectively. The grey, the cyan and the yellow balls denote C atoms, Au atoms in the outermost surface layer of the Au crystal, and the other Au atoms, respectively. The amorphous carbon cluster consists of twenty-five C atoms, and it is placed on the {311} surface of the Au crystal. The reason to use the {311} plane as the surface is that the surfaces of NPG are uneven and curved, as shown in Figs. 1c, 1e and 2, and the {311} plane is a low-index plane that can give an uneven surface. This consideration has been experimentally proven by previously reported works[1]. The IS is the

configuration of the carbon cluster adsorbed on the Au surface. In the FS, an Au atom is indicated by a red circle, and it strongly interacts with the carbon cluster, by which it has been distant from the Au surface. To reach the FS, the IS needs to evolve firstly into the TS and then into the FS. The energy change from the IS to the TS is the activation energy (E_a), whose value here is calculated to be 0.98 eV. This value is easily achievable under the actual reaction conditions, such as the high temperatures of 580 and 346 °C[2]. Therefore, the TS and thus the FS are easily reachable, because the energy of the FS is always lower than that of the TS.

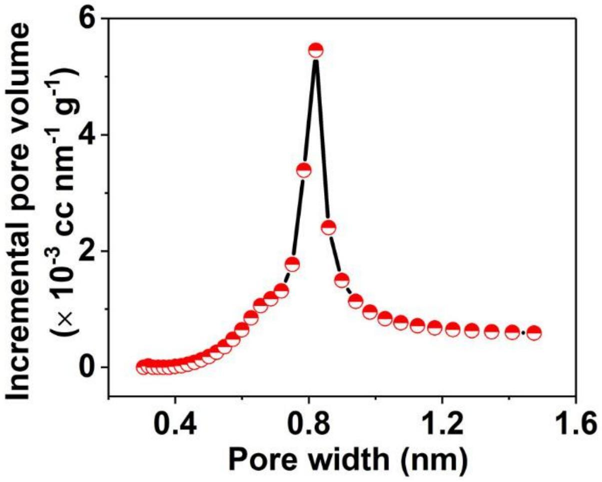

Supplementary Figure 10 Size distribution of sub-nano pores in the amorphous carbon layers, detected by CO_2 adsorption-desorption. Within the scope of our present study, NPG catalyzes CH_4 decomposition in a highly dynamic way. The deposition of amorphous carbon layers is accompanied by tremendous H_2 gas evolution, which results in the formation of multi-modal porous structure in the carbon layers. CO_2 and N_2 gas adsorption-desorption measurements (Fig. 3a and Supplementary Fig. 10) demonstrate the existence of both sub-nano pores and nanopores in the carbon layers. These pores can serve as efficient channels for mass transportation, which are key to the observed co-catalysis behavior in our system. Actually, Fig. 2 indicates that after the NPG surface was fully covered by amorphous carbon layers, the surface still continuously disintegrated, making NPG ligaments continuously slim. This phenomenon manifests that the pores truly worked as efficient channels for mass transportation and the catalytic reaction still occurred on the NPG surface that was covered by the amorphous carbon. Thus, no poisoning effect took place on the NPG surface in our work.

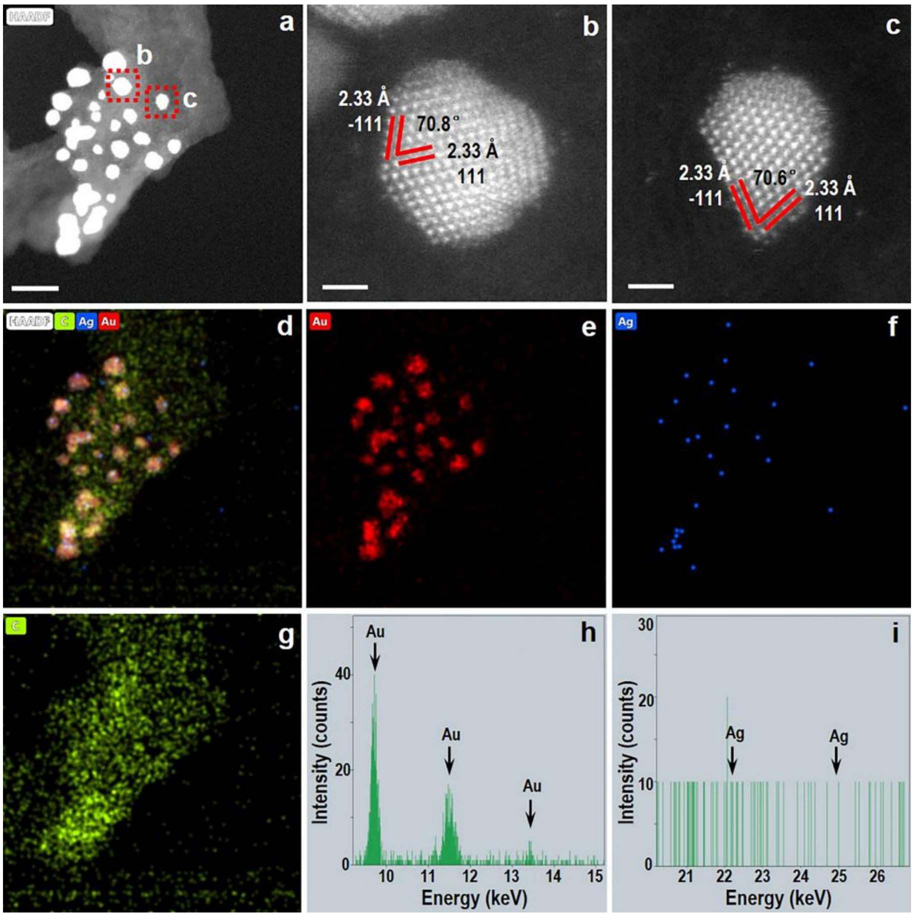

Supplementary Figure 11 HAADF images and their EDS results of some Au nanoparticles from an NPG sample through the CH_4 pyrolysis. **a**–**c** Low-magnification and atomic-resolution HAADF images of the Au nanoparticles. The images in **b** and **c** were taken from the boxed regions in **a**. Both of the lattice fringe spacings and the angles between the fringes conform to the standard Au structure, indicating that the nanoparticles are crystalline Au. **d**–**i** EDS results of these Au nanoparticles. The Ag signals in **i** are below the detecting limit, and only noise exists in the Ag map in **f**, reasonably suggesting that the Ag content in these crystalline Au nanoparticles is below the detecting limit of EDS. The images in **a** and **d**–**g** have the same scale bar of 10 nm. The scale bars in **b** and **c** are 1 nm. It should be noted that the Au nanoparticles are separate from and do not contact NPG surfaces, as shown by Fig. 3e and Supplementary Figs. 11 and 12 (for instance, the substrate containing the Au nanoparticles in Supplementary Fig. 11 is not NPG but amorphous carbon), although the

nanoparticles were formed by Au single atoms that were produced from the NPG surface disintegration. Moreover, Ag atoms in the NPG leaf contribute to the catalytic activity, as mentioned in the main text and Supplementary Fig. 26, Supplementary Note 8. Therefore, during the methane pyrolysis, Ag atoms exist on the NPG surfaces.

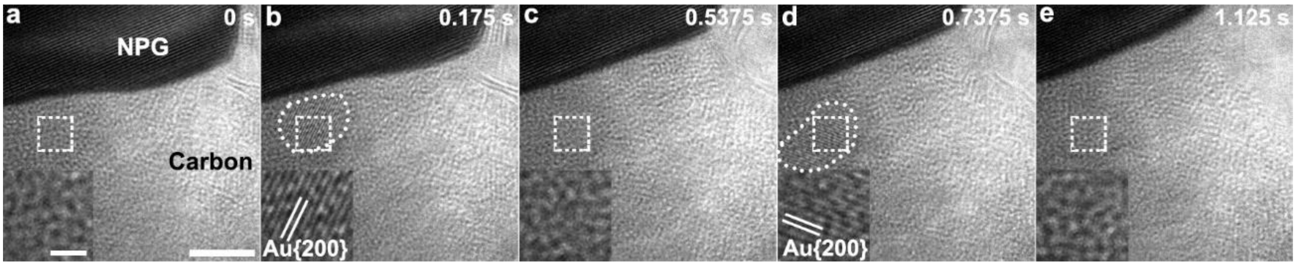

Supplementary Figure 12 Characterization of the dynamic catalysis process of Au SAs in an additional sample region. **a**–**e** HRTEM images at five different moments during the catalytic CH_4 pyrolysis reaction with all the conditions unchanged. These images are cut from Supplementary Movie 5 (see more details in Methods). They have the same scale bar. So do their insets. The moment in **a** is defined as 0 s. The insets are the close-up views of the boxed regions in the corresponding main panels, respectively. The dashed closed curves in **b** and **d** indicate the boundaries of two temporarily formed crystal nanoparticles, respectively. Scale bars: **a**, 5 nm; the inset of **a**, 1 nm.

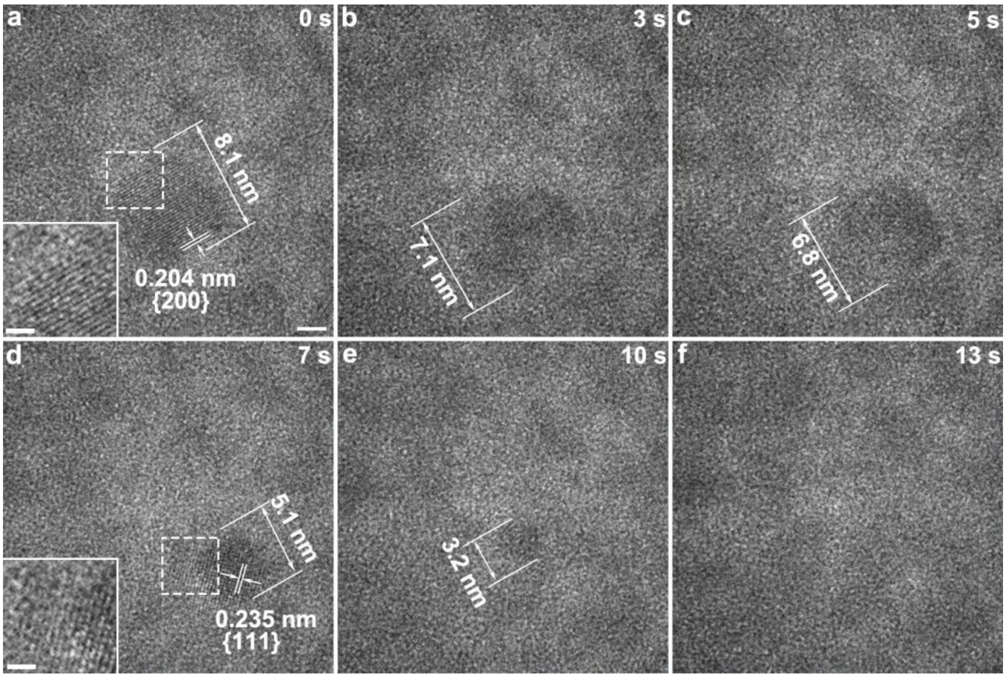

Supplementary Figure 13 Structure evolution of an Au particle during the reaction process. This Au particle initially had the diameter of 8.1 nm (**a**), then continuously overturned and disintegrated (**b**-**e**), and finally disappeared (**f**). The insets in **a** and **d** are the enlarged parts of the Au particle. Scale bars: **a**, 2 nm; the insets of **a** and **d,** 1 nm.

Supplementary Note 5 The whole system, containing amorphous carbon, single atoms, clusters and particles, is in a highly dynamic process (Supplementary movies 4 and 5). Thus, the re-orienting of the Au particles might occur during the reaction process. Therefore, we re-checked the process and recorded a new movie (Supplementary Movie 6), some of whose moments are shown in Supplementary Fig. 13**a**–**f**. The different orientations in **a** and **d** prove that re-orienting indeed occurred on the particle. But, the size reduction of the particle from 8.1 nm in **a** to 3.2 nm in **e** is rather large, and at the same moments the particle projection kept the nearly round shape. In consideration of 3D geometry, it is quite unlikely that sole re-orienting may cause such large size reduction while the nearly round projection shape maintains. Thus, it can be concluded that re-orienting and disintegrating simultaneously occurred on the particle.

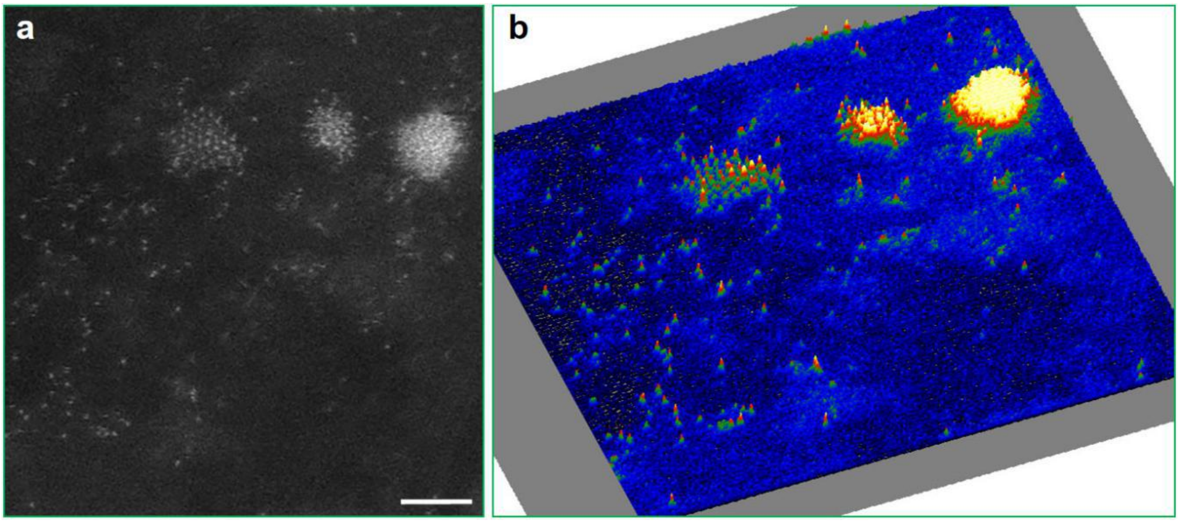

Supplementary Figure 14 a HAADF image featuring the co-existence of Au single atoms, clusters and 2D thin raft. **b** Three-dimensional (3D) rendering of the contrast intensity distribution of **a**. Scale bars: **a**, 2 nm.

Supplementary Note 6 During the methane pyrolysis, the NPG catalyst undergoes very dramatic structural evolution, during which various Au nano- or subnano-structures are dynamically observed, including Au single atoms, Au atom pairs, Au clusters (Supplementary Figs. 3**c**, **d**, 5**a**, **b**, 14), Au nanoparticles (Fig. 3**e** and Supplementary Figs. 11–14), and even 2D Au thin rafts, etc. we re-examined more than two hundred HAADF images recorded during our research, and found that 2D Au rafts indeed existed, although they were quite rare due to their metastability. Supplementary Fig. 14 shown below features the co-existence of Au single atoms, Au clusters, and a possible 2D Au raft. From the three-dimensional (3D) contrast intensity distribution of the Au structures (Supplementary Fig. 14), it can be clearly seen that the contrast intensity of the main part of the Au raft is approximately uniform, while the contrast intensities in the central parts of the Au clusters are much higher than those of their edges.

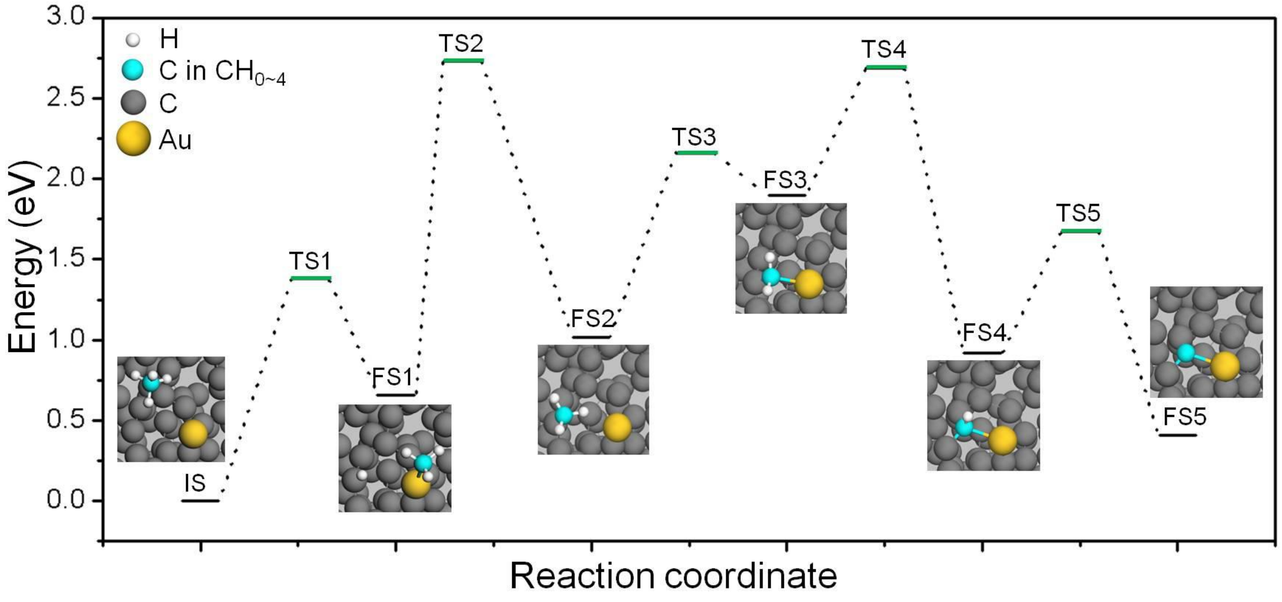

Supplementary Figure 15 DFT-calculated mechanism of the CH_4 pyrolysis reaction over an Au SA on amorphous carbon. This DFT result indicates that this mechanism contains five FSs corresponding to five elementary reactions. For instance, FS1 and TS1 are the FS and the TS of the first elementary reaction, respectively. The insets are the DFT-relaxed atomic configurations of the IS and all the FSs. The IS is a CH_4 molecule (denoted as CH_4*) adsorbed on a C atom of the amorphous carbon substrate, where an Au SA has been anchored on the substrate. FS1 indicates that CH_4* loses an H atom and becomes CH_3* adsorbed on the Au SA. In FS2, CH_3* moves to and adsorbs on a C atom of the substrate. In FS3, CH_3* loses an H atom and becomes CH_2* adsorbed simultaneously on the C atom and the Au SA. In FS4, CH_2* loses an H atom and becomes CH* adsorbed simultaneously on the C atom and the Au SA. In FS5, CH* loses an H atom and becomes C* adsorbed simultaneously on the C atom and the Au SA. The E_a values for the five elementary reactions are 1.38, 1.90, 1.14, 0.62 and 0.75 eV, respectively. These values are easily achievable under the actual reaction conditions, such as the high temperatures of 580 and 346 °C[2]. Therefore, all the TSs and thus all the FSs are easily reachable, proving the catalytic reactivity of the Au SA towards the CH_4 pyrolysis.

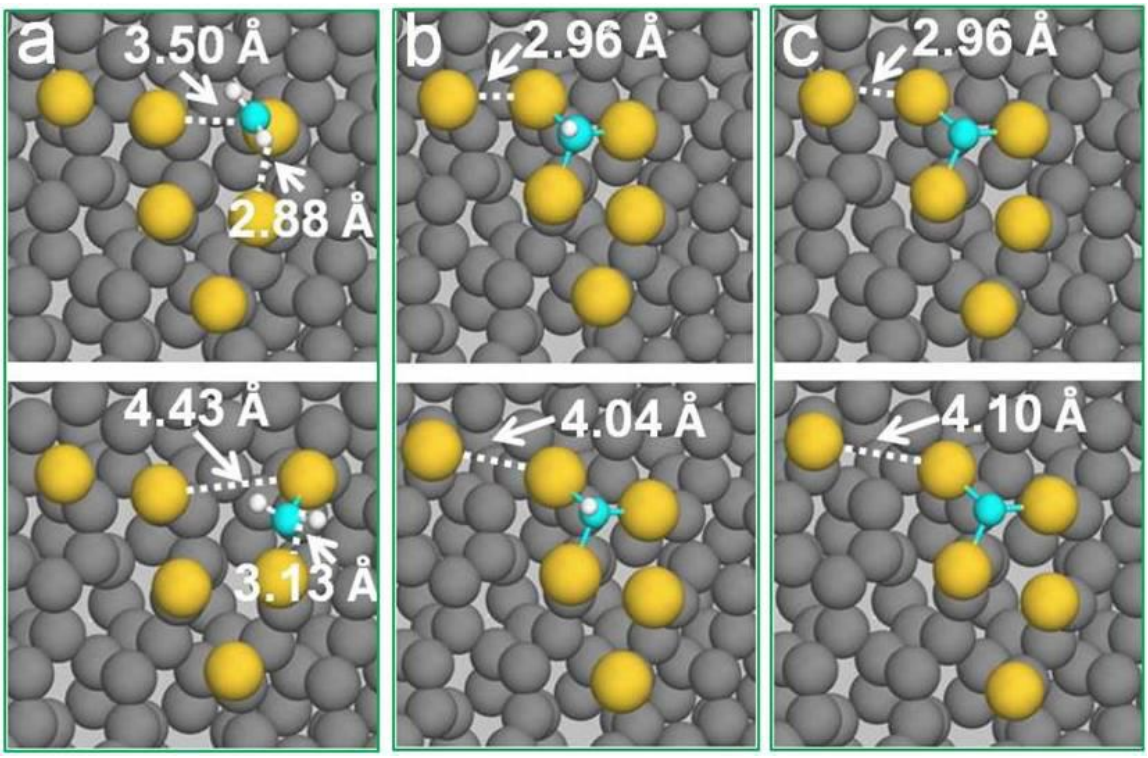

Supplementary Figure 16 DFT-relaxed atomic configurations of the ISs and the FSs of CH_2-Au/C (**a**), CH-Au/C (**b**) and C-Au/C (**c**). Each image contains two panels, of which the top and the bottom panels are the configurations of the corresponding IS and FS, respectively.

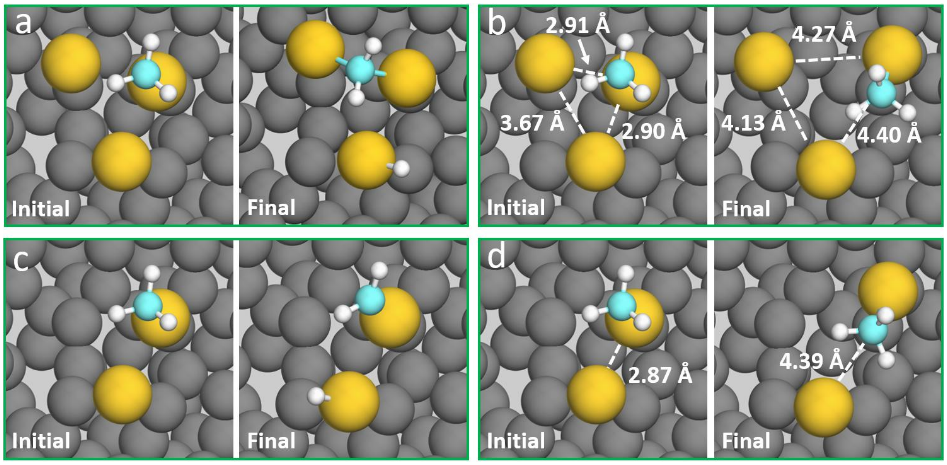

Supplementary Figure 17 DFT-calculated possible evolutions of Au_3 and Au_2 clusters during the CH_4 pyrolysis. **a** The initial and the final states of an Au_3 cluster with the adsorption and decomposition of CH_3* (CH_3 is the first intermediate of the CH_4 pyrolysis) in the case that the Au_3 cluster always exists. CH_3* becomes CH_2* (the second intermediate) in the final state. **b** The initial and the final states of the Au_3 cluster with the adsorption of CH_3* in the case that the final state of the Au_3 cluster is its breaking into three Au single atoms. **c** and **d** have the same meanings as **a** and **b**, but they are for an Au_2 cluster. Grey balls: C in amorphous carbon; cyan balls: C in CH_3* and CH_2*; yellow balls: Au; white balls: H. The C atoms in the amorphous carbon substrate are drawn purposefully to be larger than those in CH_3* and CH_2* in order to distinguish the two types of C atoms.

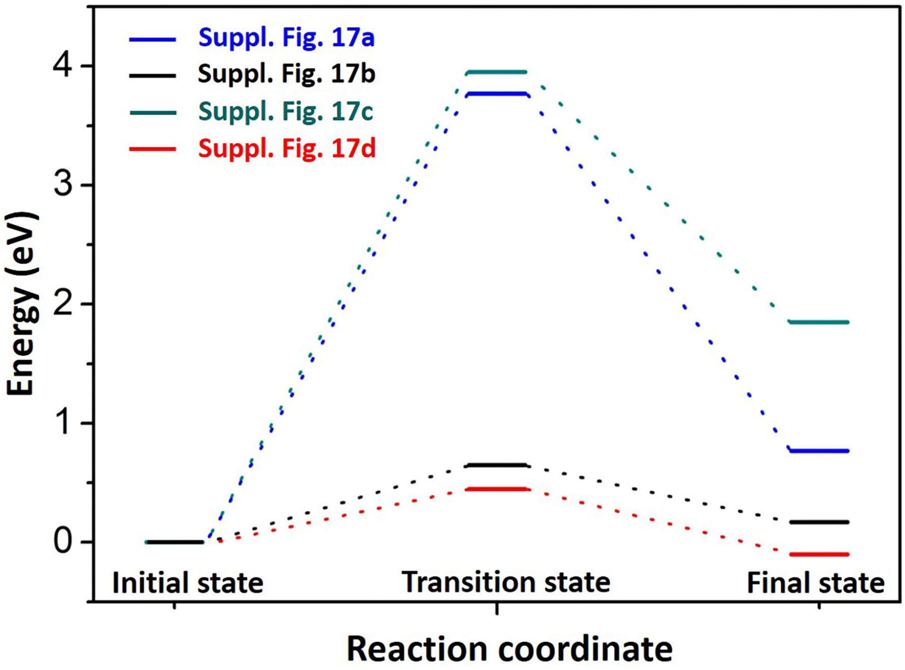

Supplementary Figure 18 Activation energies of the evolutions in Supplementary Fig. 17. The energy change from an initial state to its corresponding transition state is the activation energy (E_a). Supplementary Figs. 17**a** and 18 indicate that if the Au_3 cluster always exists, the activation energy of its catalyzing the conversion of CH_3* into CH_2* is 3.77 eV. In contrast, if the Au_3 cluster breaks into three Au single atoms under the adsorption of CH_3*, the corresponding activation energy is 0.65 eV (Supplementary Figs. 17**b** and 18), much lower than 3.77 eV. This comparison manifests that before the Au_3 cluster can catalyze the conversion of CH_3* into CH_2*, it should have broken into three Au single atoms. The Au_2 cluster has the similar result, as shown by Supplementary Figs. 17**c,d** and 18. Then, these resultant Au single atoms catalyze the proceeding and completion of the CH_4 pyrolysis, as indicated by Supplementary Fig. 15.

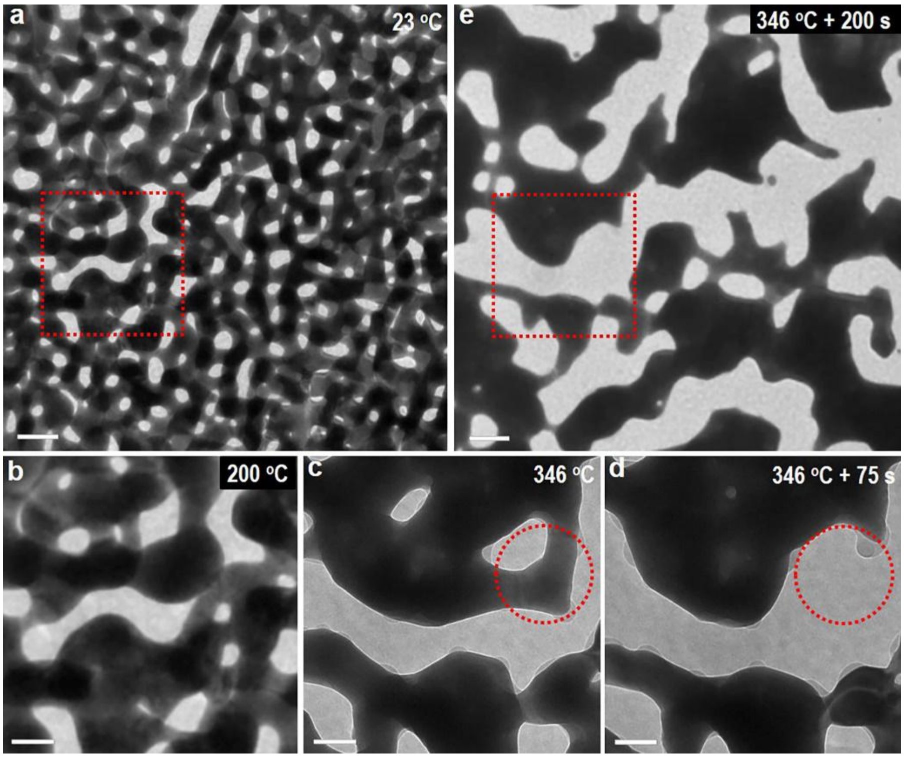

Supplementary Figure 19 Catalysis-induced structure change of NPG during the CH_4 pyrolysis. **a** Low-magnification TEM image of NPG at 23 °C. The boxed region was thereafter *in situ* characterized from 23 to 346 °C in atmospheric-pressure pure CH_4. **b**–**d** High-magnification TEM images of the boxed region at 200 °C, 346 °C (0 s) and 346 °C (75 s), respectively. The two red circles in **c** and **d** have the same position. **e** Low-magnification TEM image of NPG after the pyrolysis proceeded at 346 °C for 200 s. The boxed region in **e** has the same position as that in **a**. Scale bars: **a**, 100 nm; **b**, 50 nm; **c**, 50 nm; **d**, 50 nm; **e**, 100 nm. Comparing the circled regions in **c** and **d** shows that a ligament broke, its residual parts migrated to the adjacent ligament, and they fused together, making the adjacent ligament coarsen. The migration and the fusion are caused by the movement of the Au atoms from the broken ligament[3,4]. The images in **a**–**d** show that with the atmospheric-pressure pure CH_4, the structure of NPG underwent substantial change at 346 °C within 75 s. At the high magnification used for **b**–**d**, the boxed region was continuously irradiated by the electron beam, while other NPG parts were out of the view field and thus not or less affected by

the electron beam. When the magnification was lowered to that used for **e**, the other parts were observed to have structure changes similar to the boxed region. Moreover, comparing these results and those in Supplementary Figs. 20 and 21 indicate that the structure change of NPG at 346 °C with CH_4 is not due to the electron beam, rather, it confirms a catalytic CH_4 pyrolysis process.

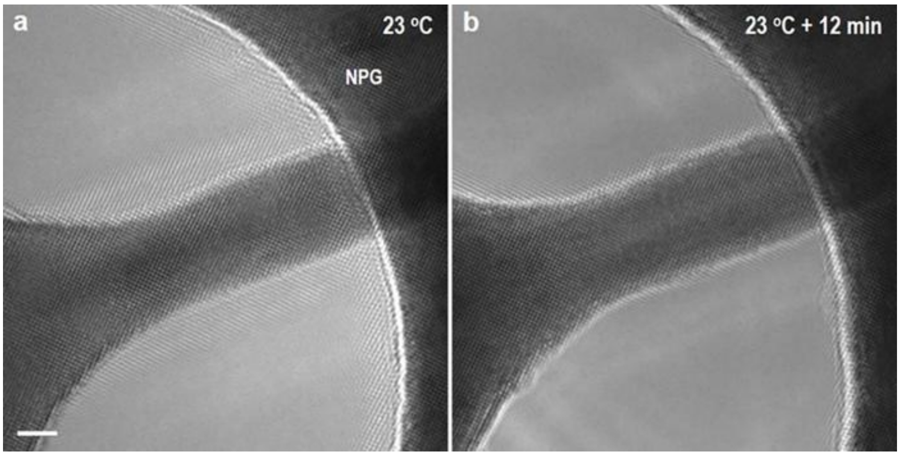

Supplementary Figure 20 HRTEM images with the highest electron dose rate of 1570 $eÅ^{-2}s^{-1}$ before (**a**) and after 12 min continuous electron beam irradiation (**b**) without CH_4. The images in **a** and **b** have the same scale bar of 2 nm. This comparison indicates that the ligament surface structure is stable under the long-time irradiation of electron beam with the highest electron dose rate. In addition, it should be noted that electron irradiation may sometimes induce amorphous carbon contamination. In our work, the as-prepared NPG samples and the TEM chamber are both clean. The images here show that the surface of NPG ligaments remains clean upon continuous 12 min electron irradiation under the HRTEM mode, without feeding the CH_4 gas. Besides, Supplementary Fig. 22 clearly proves that NPG can pyrolyze CH_4 to produce hydrogen without electron beam, indicating that the amorphous carbon on the NPG surface is induced by the CH_4 pyrolysis.

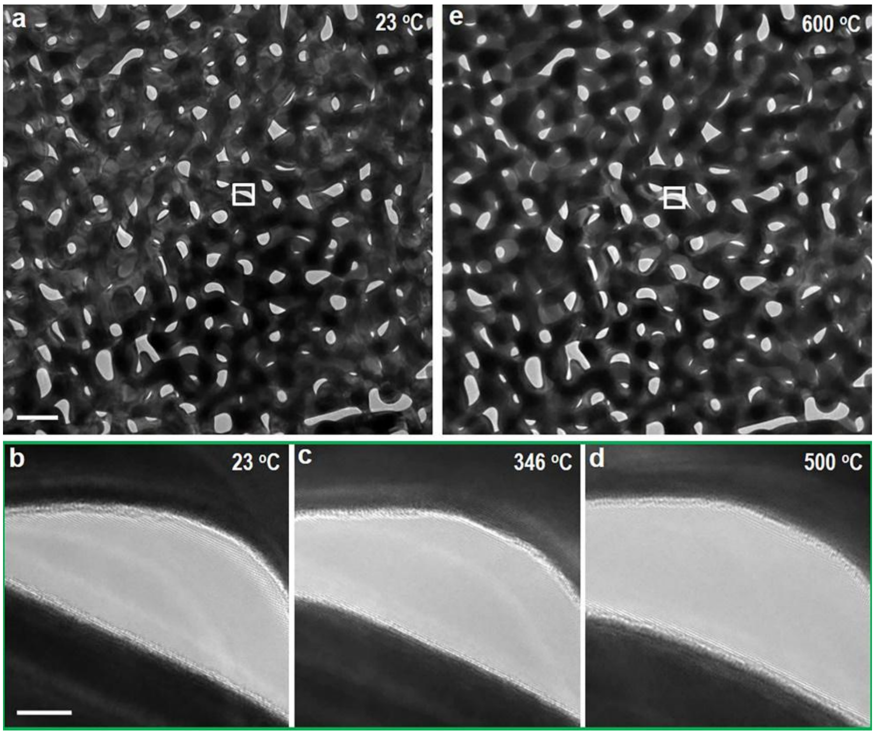

Supplementary Figure 21 Stability of NPG ligaments upon sole electron beam irradiation from 23 to 600 °C in the absence of CH_4. **a** Low-magnification TEM image at 23 °C. **b**–**d** HRTEM images of the boxed region in **a** at 23, 346 and 500 °C. **e** Low-magnification TEM image at 600 °C. The two white boxes in **a** and **e** have the same position. The images in **a** and **e** have the same scale bar of 100 nm, and those in **b**–**d** have the same scale bar of 5 nm. The *in situ* heating experiment here shows that without CH_4, the structure of nanopores and ligaments in NPG remained unchanged before the temperature reached 500 °C, as displayed by **a**–**d**. Moreover, during this heating process, the HRTEM region was irradiated continuously by the electron beam with the dose rate of 467 $eÅ^{-2}s^{-1}$ for 18 min. From 500 to 600 °C, slight coarsening of nanopores was observed due to surface diffusion enhanced by high temperature, as shown by **d** and **e**. These results indicate that no observable structure changes occurred in NPG during long-time irradiation of electron beam in heating and below 500 °C.

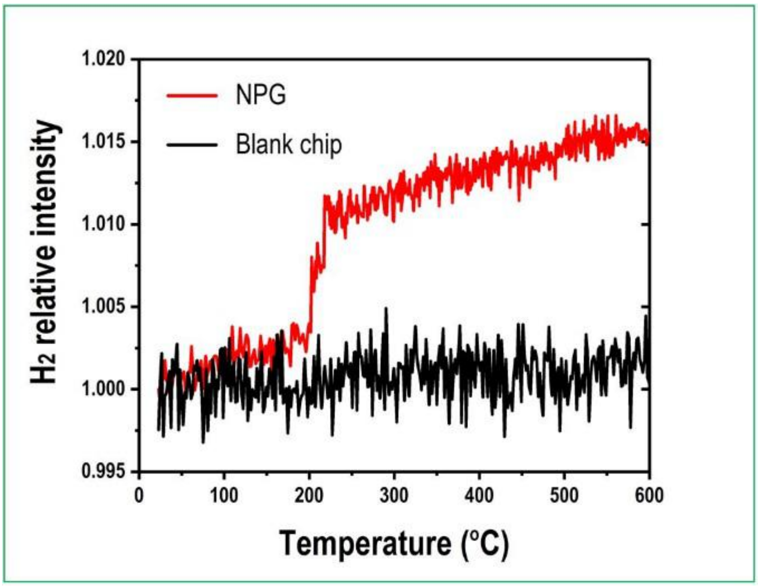

Supplementary Figure 22 Hydrogen production results of NPG and a blank chip detected by a mass spectrometer connected to the exhaust end of the *in situ* TEM holder when the electron beam was turned off. The reaction conditions here were the same as those for Supplementary Fig. 19 except keeping the electron beam off. The detection results here indicate that NPG produced hydrogen from CH_4 but the blank chip did not, clearly proving the observed catalytic performance was due to the NPG catalyst itself.

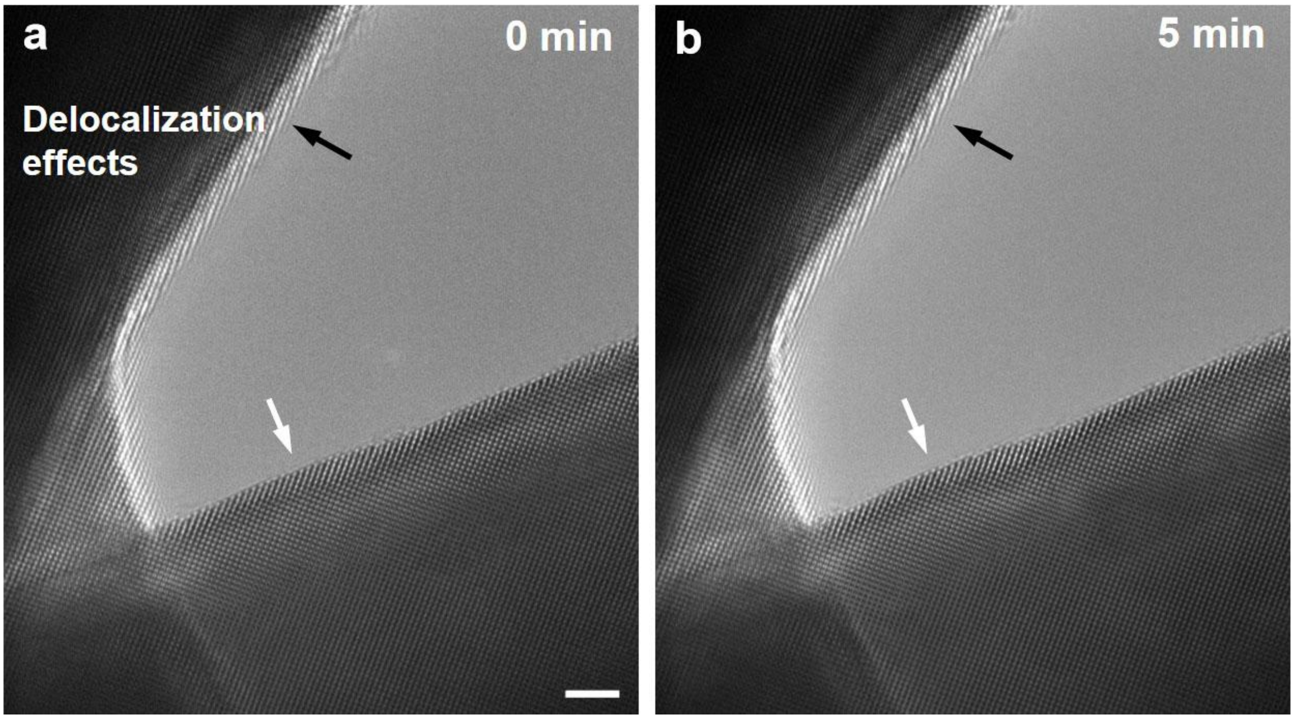

Supplementary Figure 23 HRTEM images of NPG surfaces before (**a**) and after (**b**) 5-minute continuous irradiation at 200 keV. These images were taken by FEI Themis Z with an image corrector. The delocalization effect does not and does exist on the ligament surface images indicated by the white and the black arrows, respectively. Scale bar: **a**, **b** 2 nm.

Supplementary Note 7 The delocalization effects cause lattice images to extend beyond the edges of the Au crystals and therefore mask some details that are occurring on the surfaces/interfaces. In this article, we mainly focus on the dramatic dynamic changes of NPG structure during the catalytic methane pyrolysis. For example, Fig. 2 shows that at the Au/C interfaces, the surfaces of two Au ligaments lost the thicknesses of 2.6 nm (Fig. 2**a**–**c**) and 6.1 nm (Fig. 2**d**–**f**) within 2 second. In contrast, the fringe images part extending beyond the Au edge in Fig. 2**a**–**c** was only ~1 nm thick, and the one in Fig. 2**d**–**f** was almost invisible. Therefore, such dramatic and obvious loss of the Au surfaces at the Au/C interfaces cannot be obscured by the delocalization effects. The same

phenomenon also occurred in Fig. 3**d**–**f** (namely Supplementary Movie 4) and Supplementary Movie 5.

Indeed, for too small evolutions of structures, the delocalization effects can cover up the changes. To address this issue, a TEM instrument with an image corrector (FEI Themis Z) was used. Its image corrector can eliminate the delocalization effects. As shown in Supplementary Fig. 23, the Au ligament surface indicated by the white arrow is located at the appropriate focal plane, and thus its image does not have the delocalization effect. In contrast, the surface indicated by the black arrow is located at a different height, causing that its image has the delocalization effect. Further, Supplementary Fig. 23 shows that the ligament surfaces are truly clean, and the 5-minute irradiation of the electron beam at 200 keV did not induce obvious structural changes on the surfaces, that is, the surfaces are stable. Therefore, we confirm that in the present research, it was the catalyzed pyrolysis of methane, rather than the electron beam irradiation, that induced the surface structure changes of NPG.

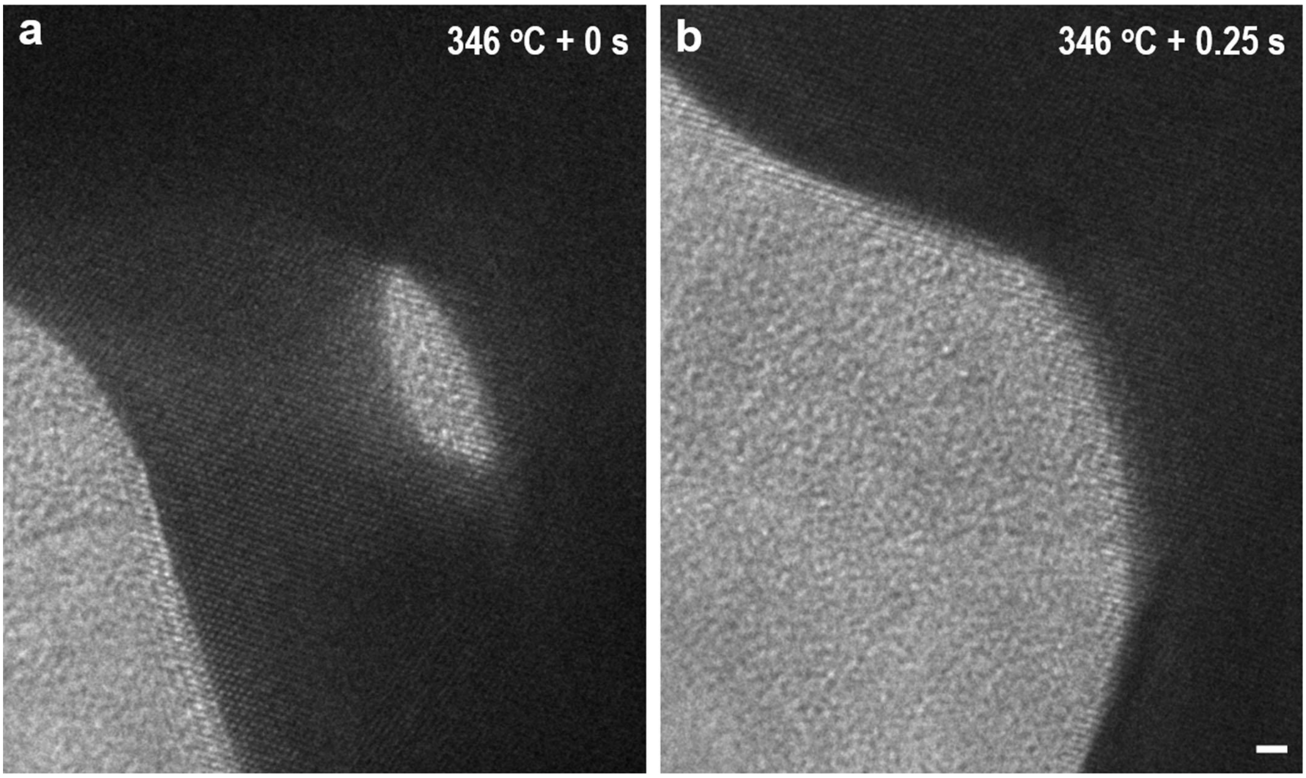

Supplementary Figure 24 HRTEM images at 0 second (**a**) and 0.25 seconds (**b**) during the CH_4 pyrolysis at 346 °C. The two images have the same scale bar of 1 nm. The electron beam was kept on. The electron dose rate used to take the images is the highest used in our experiments (1570 $eÅ^{-2}s^{-1}$). The images clearly show that once CH_4 was introduced at 346 °C, the NPG surface structure changed violently within 0.25 seconds, implying that the violent migration of Au atoms from the NPG surface took place within 0.25 seconds.

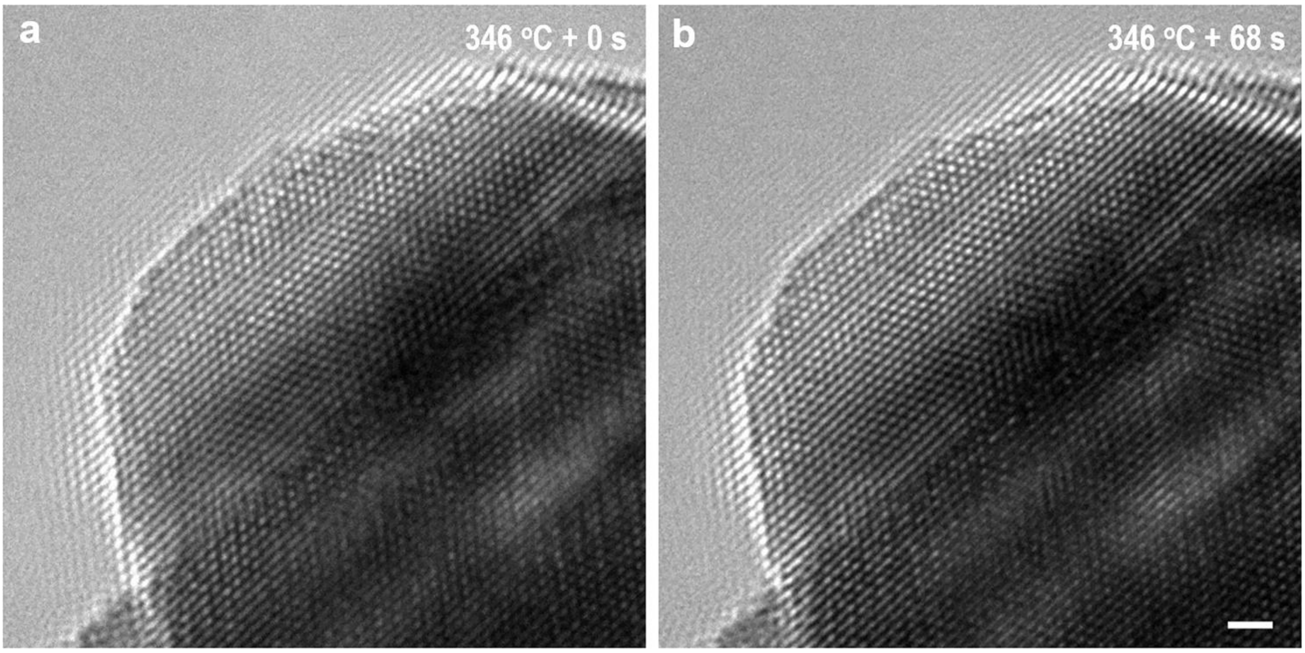

Supplementary Figure 25 HRTEM images before (**a**) and after 68 second continuous electron beam irradiation (**b**) without the CH_4 pyrolysis at 346 °C. The two images have the same scale bar of 1 nm. The electron dose rate used to take the images is the highest used in our experiments (1570 $eÅ^{-2}s^{-1}$). The irradiation duration of 68 seconds is much longer than 0.25 seconds of Supplementary Fig. 24. In contrast to Supplementary Fig. 24, the images here clearly show that when the electron beam irradiation was kept on with no CH_4 introduction, the NPG ligament surface structure was clean and stable at 346 °C for 68 seconds, which is much longer than 0.25 seconds. Moreover, Egerton, R. F. et al. have indicated theoretically that the minimum incident-electron energy to knock out Au atoms is approximately 407 keV, much higher than 200 keV of the electron irradiation used in our work[5]. Therefore, we can conclude that the migration of Au atoms from the NPG surfaces in the in-situ methane pyrolysis of our work is not induced by the electron irradiation, rather it is due to the CH_4 pyrolysis catalysis.

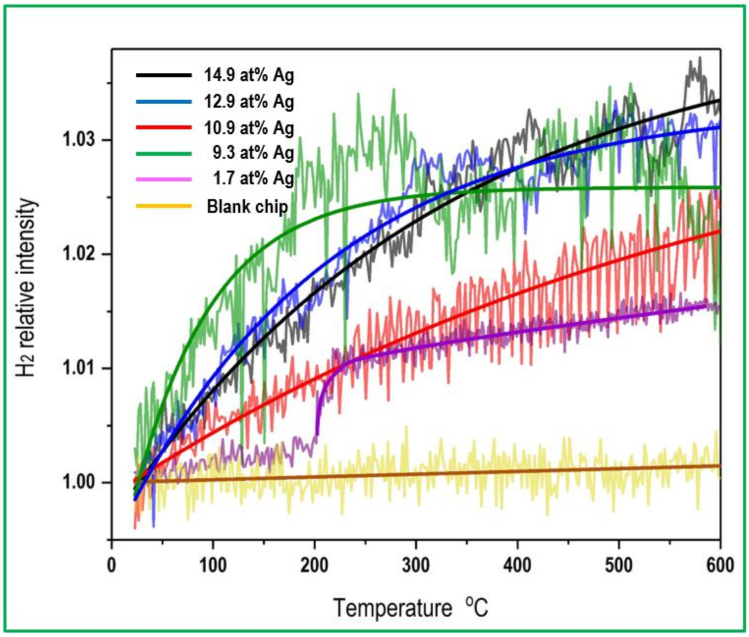

Supplementary Figure 26 Hydrogen production results during the CH_4 pyrolysis reaction using the NPG samples with different Ag contents and a blank chip without NPG. The electron beam was turned off during these experiments. The smooth lines are for eye guide.

Supplementary Note 8 The method to obtain the results here is the same as that for Supplementary Fig. 22, and the curves of the sample with 1.7 at% Ag and the blank chip are the same as those in Supplementary Fig. 22. Obviously, varying the Ag content causes different activities, and in particular the NPG sample with the least Ag content (*the* catalyst discussed in the main text) shows a distinct behavior where it becomes active only when the reaction temperature reaches 200 °C and higher. In contrast, the NPG samples with the higher Ag contents start to exhibit activities at around 50 °C. Comparisons from the different samples show that with the decrease of the Ag content, the low-temperature (<200 °C) activity decreases remarkably, and for our discussed NPG sample with the least Ag content, it shows CH_4 pyrolysis activity only above 200 °C. Actually, we selected this particular sample (with the lowest Ag content) because previous reports have proven

that CH_4 can be pyrolyzed by pure gold[6]. In our present work, we are not searching for better-activity catalysts, rather, we use the NPG (with the lowest Ag content) as a nice model system to disclose a highly dynamic process of CH_4 pyrolysis, catalyst restructuring (especially Au single atoms formation), and a unique co-catalysis process. The drastic structural change of NPG catalysts provides clear evidence of how Au ligaments restructure and how Au single atoms form, aggregate and re-disintegrate (Figs. 2 and 3 and Supplementary Movies 1, 2, 4 and 5). It is quite unlikely for such low Ag content to cause such drastic change processes, and no Ag signals were detected in the carbon layers (Supplementary Figs. 4 and 11). Therefore, for all the drastic change processes, Au reasonably plays the dominant role. It should be noted that our control experiments using NPG samples with varied residual Ag contents clearly showed that Ag atoms are capable of improving the catalyst performance and thus contribute to the catalytic activity. In our present study, the key finding is a highly dynamic structure evolution process of a nanostructured catalyst during operation. We demonstrated that NPG ligaments containing massive atoms may rapidly disintegrate during the catalytic methane pyrolysis process at 346 °C. The ligament dimension could remarkably decrease from around 13 nm to 5 nm within 2 seconds (Fig. 2d–f). Considering that the residual Ag content is relatively low (1.37 ± 0.38 at%, whose detailed data are given in the Source Data file) in our sample, it is therefore reasonable to conclude that Au plays a dominant role in this drastic structural evolution process involving massive atoms. This structural evolution includes ligament disintegration, Au single atoms formation, and Au nanoparticle formation. Meanwhile, it should also be noted that heterogeneous doping/alloying of other elements such as Ag into Au may modulate Au's catalytic properties, and in some reactions their roles may become dominant, such as CO oxidation[7]. In our present case, however, this is not the key issue for the following two reasons. First,

it has been well acknowledged that pure Au is capable for catalytic C-H activation and CH_4 pyrolysis, based on both experimental and theoretical studies[6,8], although its real-time structure evolution process during service has never been observed (which is the contribution of our present work). Second, we have supplemented the same methane pyrolysis experiments using pure Au nanoparticles, and these pure Au nanoparticles were produced by magnetron sputtering with a pure Au (>99.99%) target (see details in Methods and Supplementary Fig. 27). Our in-situ TEM and hydrogen production studies clearly proved that these pure Au nanoparticles can catalyze the CH_4 pyrolysis under the same reaction conditions (Supplementary Fig. 27). Therefore, in our work, it is reasonable to use the NPG sample with the lowest Ag content to monitor its structural evolution because the effect of Ag may be minimized.

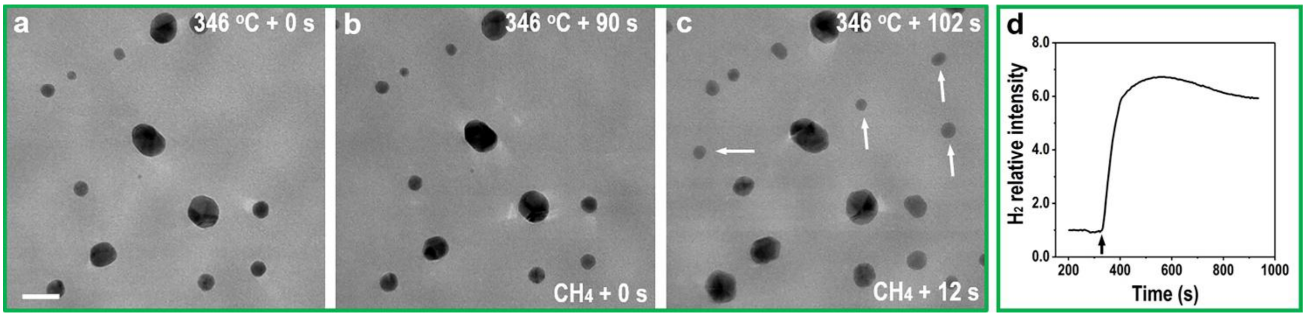

Supplementary Figure 27 Characterization of methane pyrolysis catalyzed by pure Au nanoparticles that were produced by magnetron sputtering. **a**–**c** In-situ observation of the methane pyrolysis at 346 °C. The three images in **a**–**c** have the same scale bar, 20 nm. In the first 90 seconds, there was no methane in the system, and the Au nanoparticles did not change in morphology. After the methane was introduced for 12 seconds, the morphology of the Au nanoparticles changed and small nanoparticles appeared, as indicated by the white arrows in **c**. **d** Mass spectrometry (MS) result of the hydrogen production from the methane pyrolysis catalyzed by the Au nanoparticles at 346 °C when the electron beam was turned off. The moment of introducing methane is at 330 seconds, as indicated by the black arrow. This MS result indicates that H_2 was produced by CH_4 and the Au nanoparticles. In contrast, Supplementary Fig. 22 has shown that when CH_4 was introduced into a blank chip without Au nanoparticles, no H_2 was produced under the same experimental conditions. Thus, the production experiments of hydrogen and the morphology change of the pure Au nanoparticles prove that the pure Au nanoparticles have the ability to catalyze the methane pyrolysis.

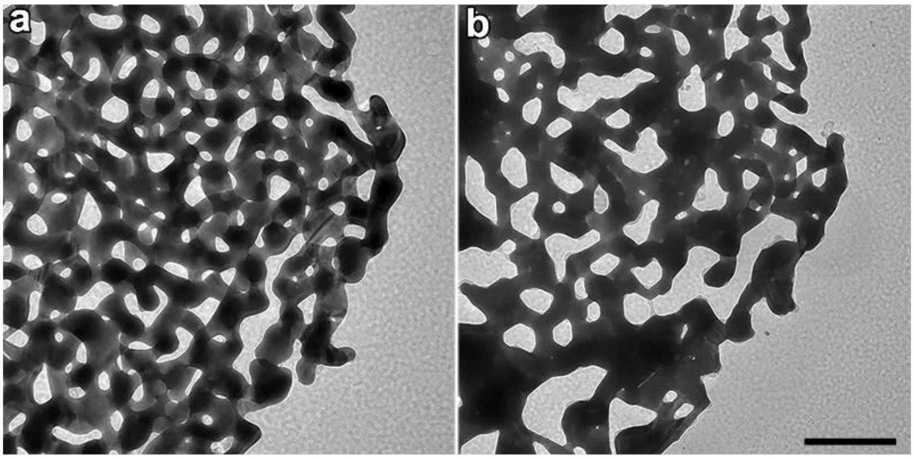

Supplementary Figure 28 Catalysis-induced structure change of NPG during the CH_4 pyrolysis. **a** TEM image of an NPG sample at 23 °C. **b** TEM image of the sample after the pyrolysis proceeded at 346 °C for 7 min. The two images have the same scale bar of 200 nm.

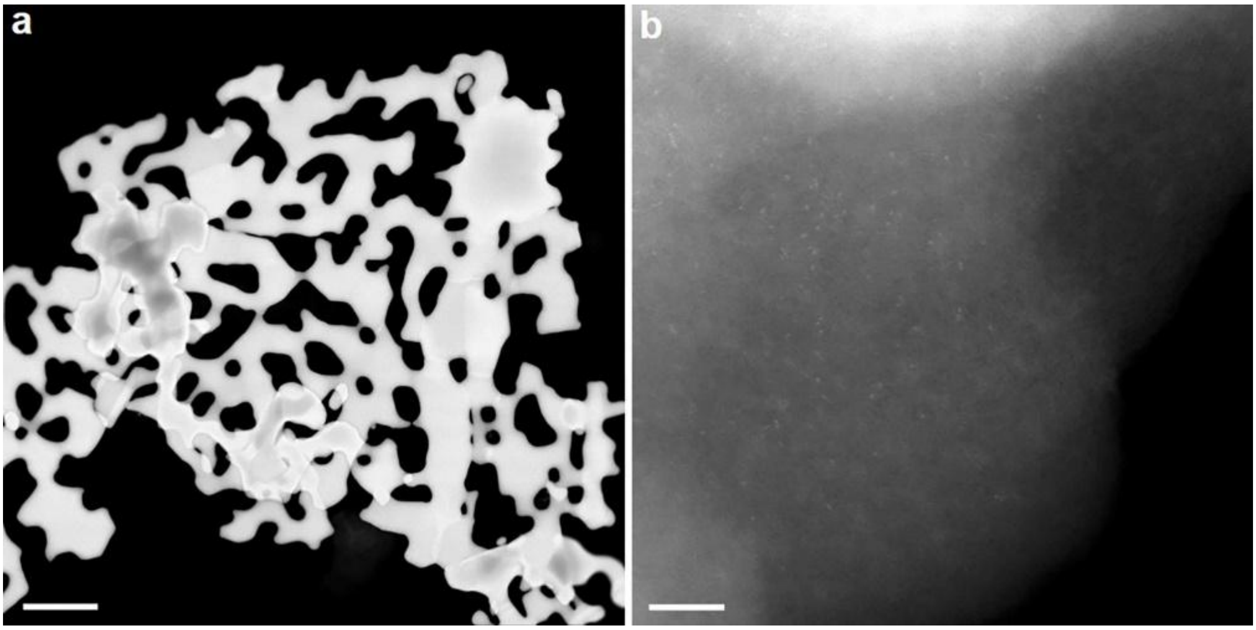

Supplementary Figure 29 Characterization of Au single atoms produced by the *in situ* CH_4 pyrolysis. **a** Low-magnification HAADF image of an NPG sample after it experienced the *in situ* CH_4 pyrolysis. **b** Atomic-resolution HAADF image of a carbon region of the sample, showing clearly Au single atoms. Scale bars: **a**, 500 nm; **b**, 5 nm. Our samples for the *in situ* study were prepared via the standard dealloying method and were not treated with any special method such as plasma treatment. After the *in situ* TEM experiments, the samples were characterized by HAADF, showing clearly the existence of Au single atoms in the carbon layers (Supplementary Fig. 29**b**). This result indicates that the Au single atoms were produced by the disintegration of the NPG surface during the CH_4 pyrolysis. Moreover, we have also prepared similar NPG samples with carbon via the *ex situ* CH_4 pyrolysis reaction. These samples were also confirmed to contain Au single atoms (Fig. 1f). By comparing both *in situ* (with electron irradiation) and *ex situ* (without electron irradiation) results, we reveal a highly dynamic process of how Au catalyzes the CH_4 pyrolysis in real space and time.

Supplementary Table 1 Calculated Activation Energies (eV) for C-H bond cleavage[9,10]

Elementary reaction	Ag_{10}	Au_{10}
$CH_4* \rightarrow CH_3*+H*$	1.54	1.42
$CH_3* \rightarrow CH_2*+H*$	1.60	1.45
$CH_2* \rightarrow CH*+H*$	1.61	1.17
$CH* \rightarrow C*+H*$	1.90	1.95

*: Surface of the catalyst

Supplementary References

1. Liu, P. et al. Visualizing under-coordinated surface atoms on 3D nanoporous gold catalysts. *Adv. Mater*. **28**, 1753–1759 (2016).
2. Young, D. C. *Computational chemistry: apractical guide for applying techniques to real-world problems* (A John Wiley & Sons Publication, New York, 2001.)
3. Fujita, T. et al. Atomic origins of the high catalytic activity of nanoporous gold. *Nat. Mater.* **11**, 775–780 (2012).
4. Fujita, T. et al. Atomic observation of catalysis-induced nanopore coarsening of nanoporous gold. *Nano Lett.* **14**, 1172–1177 (2014).
5. Egerton, R. F., McLeod, R., Wang, F. & Malac, M. Basic questions related to electron-induced sputtering in the TEM. *Ultramicroscopy* **110**, 991–997 (2010).
6. Lang, S. M., Bernhardt, T. M., Chernyy, V., Bakker, J. M., Barnett, R. N. & Landman, U. Selective C–H bond cleavage in methane by small gold clusters. *Angew. Chem. Int. Ed.* **56**, 13406–13410 (2017).
7. Kamiuchi, N. et al. Self-activated surface dynamics in gold catalysts under reaction environments. *Nat. Commun*. **9**, 2060 (2018).
8. Zhong, D. et al. Linear alkane polymerization on a gold surface. *Science* **334**, 213–216 (2011).
9. Au, C. T., Ng, C. F. & Liao, M. S. Methane dissociation and syngas formation on Ru, Os, Rh, Ir, Pd, Pt, Cu, Ag, and Au: a theoretical study. *J. Catal.* **185**, 12–22 (1999).
10. Liao, M. S. & Zhang, Q. E. Dissociation of methane on different transition metals. *J. Mol. Catal. A Chem.* **136**, 185–194 (1998).
