# Supplementary material for: Dynamic co-catalysis of Au single atoms and nanoporous Au for methane pyrolysis
Source: Nat Commun. 2020 Apr 21;11:1919. doi: 10.1038/s41467-020-15806-8 (PMC7174348; doi:10.1038/s41467-020-15806-8)
Supplement: Supplementary file 3 — Description of Additional Supplementary Files [file 41467_2020_15806_MOESM3_ESM.pdf]

Description of Additional Supplementary Files

File Name: Supplementary Movie 1
Description: *In situ* dynamic process of the CH_4 pyrolysis reaction on an NPG surface region with positive curvature

File Name: Supplementary Movie 2
Description: *In situ* dynamic process of the CH_4 pyrolysis reaction on an NPG surface region with negative curvature

File Name: Supplementary Movie 3
Description: *In situ* observation of an amorphous carbon layer after it was produced and the CH_4 flow was stopped with the other conditions of the CH_4 pyrolysis reaction unchanged

File Name: Supplementary Movie 4
Description: *In situ* dynamic process of an amorphous carbon layer during the CH_4 pyrolysis reaction with all the conditions unchanged

File Name: Supplementary Movie 5
Description: *In situ* dynamic process of a second amorphous carbon layer during the CH_4 pyrolysis reaction with all the conditions unchanged

File Name: Supplementary Movie 6
Description: *In situ* dynamic process of the simultaneous disintegrating and overturning of an Au particle during the CH_4 pyrolysis reaction with all the conditions unchanged
